# Effect of cellular senescence on the P2Y-receptor mediated calcium response in trabecular meshwork cells

**Published:** 2007-10-16

**Authors:** Jessica Chow, Paloma B. Liton, Coralia Luna, Fulton Wong, Pedro Gonzalez

**Affiliations:** Department of Ophthalmology, Duke University, Durham, NC

## Abstract

**Purpose:**

The objectives of this study were to evaluate the potential release of ATP that is mediated by mechanical stress on trabecular meshwork (TM) cells, to identify the specific P2Y receptors mediating the ATP response, and to determine whether cellular senescence might interfere with the P2Y receptor-mediated calcium response, thus contributing to the loss of physiologic TM function in aging and primary open angle glaucoma (POAG).

**Methods:**

Experiments were conducted using primary cultures of porcine TM cells. Cyclic mechanical stretch (10% stretching/second) was generated using the Flexcell system. ATP release and ectoATPase activity induced by mechanical stress were measured using a luciferin/luciferase assay. Replicative senescence was induced by passing the cells 18 times at a 1:2 split ratio and confirmed by the presence of senescence-associated β-galactosidase (sa-β-gal) and autofluorescence. For calcium imaging, cells were plated on gelatin-coated coverslips, bathed in calcium Ringer's solution, and loaded with fluo-4 (5 μM) for 1 h. Agonists of P2Y1 (ADP) and P2Y2/P2Y4 (ATP, UTP) receptors at 10 μM or 100 μM concentrations were added to the bathing medium. Relative changes in cytosolic calcium concentration as a function of time were measured by fluorescent microscopy and reported as peak amplitudes of fluo-4 fluorescence normalized to baseline values (ΔF/Fo).

**Results:**

Mechanical stress induced an increase in ATP release from TM cells (258%±23% at 15 min, 188%±11% at 30 min, and 900%±203% at 1 h; p<0.017, n=4) as well as an increase in ectoATPase activity present in the extracellular media during the first 15 min of stress (57%±15%, p=0.011, n=4). The P2Y receptor agonists listed above induced a concentration-dependent rise in intracellular calcium in the TM cells. The peak amplitude, ΔF/Fo, was 1.07±0.12 (n=3) for 10 μM ADP, 2.59±0.33 (n=6) for 100 μM ADP, 1.21±0.64 (n=12) for 10 μM UTP, 3.22±2.0 (n=12) for 100 μM UTP, 0.88±0.40 (n=9) for 10 μM ATP, and 1.37±0.61 (n=25) for 100 μM ATP. Cells at passage 18 showed significantly lower levels of intracellular calcium induced by ATP (36%), UTP (34%), and ADP (52%) compared to cells at passage 2, independent from any changes in P2Y receptor changes in expression.

**Conclusions:**

The ability to release ATP in response to mechanical stress and the presence of functional P2Y receptors in TM cells suggest a novel mechanism by which TM cells could sense and respond to changes in intraocular pressure (IOP). In addition, the decrease in P2Y receptor-mediated calcium responses observed in senescent TM cells suggests that the disregulation of calcium homeostasis in senescence may contribute to the alterations of the TM in aging and POAG.

## Introduction

Aqueous humor outflow resistance through the trabecular meshwork (TM) is a critical parameter for the maintenance of normal levels of intraocular pressure (IOP). Increased resistance to outflow through the TM arises both as a consequence of the normal aging process and in the pathology of primary open angle glaucoma (POAG). However, the specific mechanisms that modulate physiologic levels of outflow resistance as well as those involved in the increased resistance associated with age and POAG are not well understood.

TM cell volume appears to be an important factor in aqueous humor outflow resistance. Cell swelling and shrinking has been demonstrated to affect outflow facility [1,2]. P2Y receptors are G_q_-protein coupled receptors that respond to extracellular nucleotides such as ATP, ADP, and UTP by increasing intracellular calcium through the IP_3_-mediated pathway. Changes in cytosolic calcium can affect cell volume regulation by activating Ca^2+^-dependent ion channels in cellular membranes and thus alter ion and water outflow. The presence of functional P2Y receptors and their involvement in cell volume regulation have been reported in TM cells [2].

A role for P2Y receptors in the modulation of IOP is suggested by the reported observation that selective P2Y1 agonists induce outflow facility increases in perfused anterior segments from bovine eyes, and this effect is prevented by selective P2Y1 receptor antagonists [3]. TM cells experience mechanical deformation as a result of increased IOP. In addition, in vivo observations have shown that the TM is constantly subjected to cyclic mechanical stress [4-9]. Mechanical stress is known to induce the release of a major P2Y receptor agonist, ATP, in numerous cell types including vascular endothelial cells, human tendon cells, and subepithelial fibroblasts [10-15]. Similarly, P2Y receptor-mediated cell volume regulation could be initiated in response to the increased mechanical stress associated with elevated IOP. The potential stress-induced release of ATP might therefore influence TM cell volume and thus, basal levels of outflow resistance. However, a cause and effect relationship between mechanical stress on TM cells and ATP release from TM cells has not yet been demonstrated.

The potential involvement of P2Y-mediated calcium signaling in both the response of the TM to IOP elevations and the maintenance of basal levels of outflow resistance could also be relevant toward understanding the increase in aqueous humor outflow resistance associated with aging and POAG. Cellular senescence has been hypothesized to contribute to organismal aging and to the pathology of several age-related diseases such as atherosclerosis and osteoarthritis [16-18]. We have previously shown that TM cells from POAG donors express significantly higher levels of a well characterized marker for cellular senescence, senescence-associated β-galactosidase (sa-β-gal), compared to age-matched normal controls [19]. Cellular senescence is known to be associated with a break down in calcium homeostasis [20]. Such disregulation could limit the ability of the TM to respond to P2Y agonists and therefore might contribute to a failure of TM cells to modulate physiologic levels of outflow resistance in response to IOP changes in both aging and POAG.

The objectives of this study were to: (1) evaluate the potential release of ATP that is mediated by mechanical stress on TM cells, (2) identify the specific P2Y receptors mediating the ATP response, and (3) determine whether cellular senescence might interfere with the P2Y receptor-mediated calcium response and thus contributing to the loss of physiologic TM function in aging and POAG.

## Methods

### Primary trabecular meshwork cell cultures

Porcine eyes were obtained from a local slaughterhouse. Each intact porcine eye was soaked in complete growth medium, Dulbecco's Modified Eagle Medium (DMEM), with 20% heat-inactivated fetal bovine serum (FBS) for 15 min at room temperature. The TM was dissected and digested with 2 mg/ml of collagenase for 1 h at 37 °C. The tissue explants were placed in 2% gelatin-coated, 35 mm dishes and cultivated in DMEM with 20% FBS (Invitrogen, Carlsbad, CA) and 50 mg/ml gentamicin (Invitrogen). After passage one, serum was reduced to 10% for routine cultivation. Cell lines were subcultivated 1:2 when confluent. Cell cultures were maintained and propagated at 37 °C in an atmosphere of 5% CO_2_ and 5% O_2_.

### Cyclic mechanical stress application in cell culture

Porcine TM cells at passage 3 were plated on type I collagen-coated flexible silicone bottom plates (Flexcell, Hillsborough, NC) and grown to confluence. Culture medium was replaced by serum-free DMEM for 3 h, then cells were subjected to cyclic mechanical stress (10% stretching, 1 cycle/second) using a computer-controlled, vacuum-operated FX-3000 Flexercell Strain Unit (Flexcell, Hillsborough, NC). Control cells were cultured under the same conditions, but no mechanical force was applied.

### ATP release

Thirty min before stretching, 100 μM of the specific ecto-ATPase inhibitor ARL-67156 (Sigma, St. Louis, MO) and 100 μM of the P2 receptor antagonist reactive blue 2 (Sigma) were added to the cell cultures to reduce ATPase activity. Supernatant fluid samples for ATPase release measurements were collected from TM cell cultures with (experimental) or without (control) stretching at times 0, 15 min, and 30 min. Samples were kept on ice to decrease ecto-ATPase activity and centrifuged to remove cell debris, and ATP measurements of the supernatant were determined by luciferin-luciferase assay (ATP determination kit; Molecular Probes, Carlsbad, Ca) using a TD-20/20 luminometer (Turner Designs, Sunnyvale, CA). Duplicate samples were collected from each well and the results were averaged. The experiment was repeated in triplicate to verify results.

### Ecto-ATPase activity

Before stretching was initiated, 10 μM ATP was added to cell cultures. Fifty microliters of the supernatant was collected at times 0, 15 min, and 30 min. Samples were collected as above and centrifuged. Relative ATP concentrations were then determined by luciferin-luciferase assay.

### Protein extraction and western blot

Cells were washed twice in PBS and lysated in 2X Laemmli buffer. Protein extracts were boiled for 10 min and centrifuged at 16,000 g. Protein concentration was determined by the Bradford protein assay. Proteins were separated by 10% SDS–PAGE and transferred to PVDF membrane (BioRad, Hercules, CA). Membranes were blocked with 5% non-fat dry milk and incubated overnight with specific antibodies against P2Y1, P2Y2, and P2Y4 receptors (Alomone Laboratories, Jerusalem, Israel). Immunoreactive bands were detected by incubation with a secondary antibody conjugated to horseradish peroxidase and chemiluminescence substrate (SuperSignal, West Pico; Pierce, Rockford, IL). Molecular mass was estimated using Totallab image software (Nonlinear Dynamics, Durham, NC).

### Reverse-transcription polymerase chain reaction analysis

After removing the culture medium, cells were immediately immersed in RNAlater^TM^ (Qiagen, Valencia, CA) to preserve RNA integrity. Total RNA was isolated from porcine TM cultures using an RNase kit (Qiagen) according to the manufacturer's protocol and treated with DNase. RNA yields were determined using Ribogreen fluorescent dye (Molecular Probes).

First strand cDNA was synthesized from 0.5 μg total RNA by reverse transcription using an oligo dT primer and Superscript II reverse transcriptase (Invitrogen) according to the manufacturer's instructions. Real-time polymerase chain reactions (PCR) were performed in a 20 μl mixture composed of 1 μl of the cDNA preparation, 1X iQ SYBR Green Supermix (BioRad), and 500 nm of each primer in an iCycler iQ system (BioRad) using the following PCR parameters: 95 °C for 5 min followed by 50 cycles of 95 °C for 15 s, 60 °C for 15 s, and 72 °C for 15 s. The fluorescence threshold value (C_T_) was determined using the iCycle iQ system software. The absence of nonspecific products was confirmed by both the analysis of the melt curves and by electrophoresis of the amplified products in 3% Super AcrylAgarose gels. β-Actin was used as an internal standard of mRNA expression. The sequences of the primers used for the amplifications were as follows: P2Y1F 5′-CCT GGA CAA CTC ACC TCT GA-3′, P2Y1R 5′-GGC CCT CAA ATT CAT TGT TT-3′, P2Y2F 5′-CAC GGA ACT GAC ACG AAG AG-3′, P2Y2R 5′-CTC CTA CAG CGG ATG TCT T-3′, P2Y4F 5′-TAT GCC GTT GTC TTT GTG CT-3′, P2Y4R 5′-GCG ACA GGA CGT ACA AAG TG-3′, P2Y11F 5′-AGA GTC TAT GGC CTG GTG CT-3′, and P2Y11R 5′-AGC ACT ATC ACG TGC AGC TT-3′.

### Isolation of genomic DNA from porcine trabecular meshwork cells

Porcine TM cells at passage 3 were trypsinized and resuspended in 10 mM Tris-HCl (pH 8.0), 10 mM EDTA. SDS, and proteinase K were added to a final concentration of 0.5% and 200 μg/ml, respectively. After incubation at 55 °C for 2 h, NaCl was added to a final concentration of 0.2 M. The mixture was extracted twice with phenol:chloroform (1:1) and once with chloroform alone, then placed in a 55 °C water bath to evaporate the chloroform. RNase A (DNase and protease free) was added to a final concentration of 25 μg/ml, and the mixture was incubated for 1 h at 37 °C, followed by extraction twice with phenol:chloroform (1:1) and once with chloroform alone. Genomic DNA was precipitated with ethanol and centrifuged at 10,000x g for 5 min to form a DNA pellet, which was resuspended in 10 mM Tris-HCl (pH 8.0) and 1 mM EDTA. Using reverese-transcription polymerase chain reaction (RT–PCR) methods, porcine TM cell genomic DNA was probed for the P2Y4 receptor signal to verify the functionality of the designed primer.

### Determination of intracellular calcium

The optical setup for intracellular Ca^2+^ measurements consisted of a Fluoview FV300 laser scanning confocal microscope (Olympus, Tokyo, Japan). Confocal images were acquired at an emission wavelength of 505 nm after excitation at 488 nm in time intervals ranging from 0.5 to 5.0 s. The fluorescence intensity of selected regions of interest was plotted against time. All the acquired images were stored using Fluoview 4.2 with Tiempo (Olympus) software and manipulated in Microsoft Excel (Microsoft, Redmond, WA).

TM cells were loaded with 5 μM of fluo-4 AM (Molecular Probes) for 1 h at 37 °C. The coverslips were set at the bottom of a glass chamber connected to a perfusion reservoir, which allowed the sequential application of different solutions to the specimen. For pharmacological examination of the P2Y receptor subtype-mediating cell responses, P2Y receptor agonists and antagonists were added at varying concentrations to the perfusion medium (Ringer's solution with or without calcium) and applied to the specimen. Relative changes in cytosolic calcium concentration as a function of time were measured by fluorescent microscopy and reported as peak amplitudes of fluo-4 fluorescence normalized to baseline values (ΔF/F_0_).

### Model for replicative senescence

Porcine TM cells were passaged by trypsinization at a split ratio of 1:2. Comparisons were conducted between cell cultures at passage 2 and passage 18. Cells were plated on gelatin-coated coverslips and were incubated for two days in preparation for experiments.

### FACS analysis of endogenous fluorescence

Porcine TM cells were washed with PBS, digested with 0.25% trypsin solution (Invitrogen), centrifuged at 120x g, and resuspended in 1% paraformaldehyde at 10^7^ cells/ml for analysis in a FACS analyzer (Becton Dickinson, Franklin Lakes, NJ) at 563–607 nm. Lipofuscin-specific fluorescence was quantified by flow cytometry measuring cellular autofluorescence in the yellow-green range of spectrum (563–607 nm).

### Quantification of sa-β-gal

To measure sa-β-gal activity, alkalinization of the lysosomal compartment was induced by treating cell monolayers with 300 μM chloroquine for 1 h at 37 °C under 5% CO_2_. Cells were then trypsinized, resuspended in 50 μl of PBS, and warmed at 37 °C for 5 min. A volume of 50 μl of pre-warmed 2 mM fluorogenic substrate C_12_FDG (Molecular Probes) was added to the 50 μl aliquot of cells. The cells and C_12_FDG were rapidly mixed and immediately returned to a 37 °C bath. After 1 min of incubation, the cells were resuspended in cold PBS and immediately analyzed by FACScan [21]. FDG fluorescence was detected in the FL1 channel. FACS was performed using the same settings as for GFP quantification. The mean fluorescence values obtained for each cell culture population provide a quantitative reading of the sa-β-gal staining.

## Results

### Mechanical stress induces the release of ATP and increases ecto-ATPase activity in TM cells induced by mechanical stress

**Figure 1:**
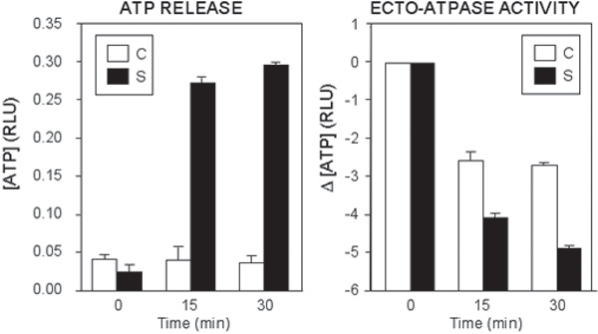
Induction of ATP release and ecto-ATPase activity after mechanical stress in trabecular meshwork cells

Cyclic mechanical stress of TM cells resulted in a significant increase in the extracellular levels of ATP in the presence of ATPase inhibitors, ARL-67156 and reactive blue 2 (Figure 1A). Ecto-ATPase activity in the cell culture media from mechanically stressed cells was also significantly higher than that of the nonstressed controls (Figure 1B).

ATP released by TM cells in cyclically stretched and nonstretched control cultures was measured using the luciferin-luciferase assay in presence of the ecto-ATPase inhibitors, ARL-67156 and reactive blue 2. Cyclically stretched cells released a significantly higher amount of ATP compared to the controls (p<0.05). To evaluate the potential increase of ecto-ATPase induced by cyclic mechanical stress, 10 μM ATP was added to the culture medium 30 min before stretching to allow cells to equilibrate after gentle shaking. Extracellular ATP was degraded at a significantly higher rate in the medium from stretched cell cultures compared to the medium from unstressed controls, indicating an increase in ecto-ATPase activity with mechanical stress (right). ATP concentration in the media is represented as relative light intensity units (RLU) and was generated in three independent experiments.

### P2Y receptor-mediated calcium mobilization in porcine trabecular meshwork cells

**Figure 2:**
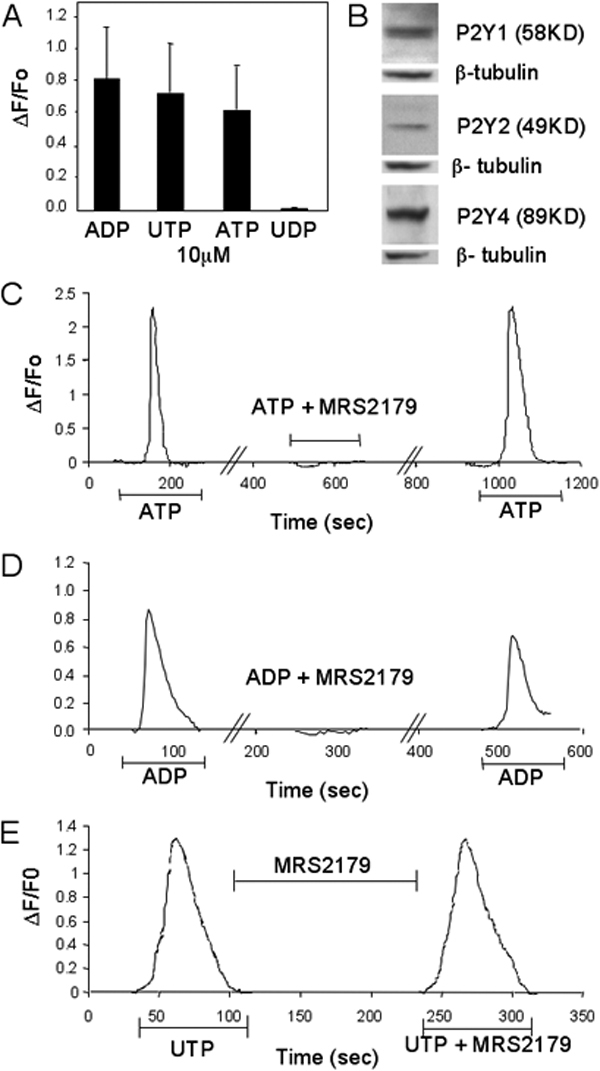
P2Y-mediated calcium response in trabecular meshwork cells

ATP, ADP, and UTP induced a significant rise in [Ca^2+^]_i_ in TM cells at passage 2 (Figure 2A). Intracellular calcium concentrations peaked within 20–30 s and returned to baseline values within 80 s of the initial rise. The calcium response exhibited a complex morphology with oscillations of varying frequencies. UDP did not induce an increase in [Ca^2+^]_i_.

Treatment of porcine TM cells with ATP, UTP, and ADP induced an increase in intracellular calcium (ΔF/F_0_) with potency occurring in the following order: ADP>UTP>ATP. UDP did not affect intracellular calcium (**A**). The expression of P2Y1, P2Y2, and P2Y4 receptors in cultured porcine cells was confirmed by WB analysis (**B**). Selective inhibition of the P2Y1 receptor with MRS2179 reversibly blocked the response to ATP and ADP (**C**, **D**). However, the calcium response to UTP in porcine TM cells was not affected by the presence of the specific P2Y1 receptor antagonist MRS2179 (**E**). The results are representative of three independent sets of experiments.

The magnitudes of the calcium responses to ATP, ADP, and UTP were not significantly affected by incubation for 10 min in low calcium Ringer's solution with EGTA (p-value 0.4038), suggesting that the increase in [Ca^2+^]_i_ resulted from the release from intracellular stores. While P2Y receptor activation increases intracellular calcium concentration primarily through mobilization of calcium from intracellular stores, P2X receptor activation increases intracellular calcium concentration largely through activation of voltage-dependent calcium influx pathways [22]. Therefore, the observed increase of intracellular calcium mediated by ATP, ADP, and UTP is not likely to be mediated by P2X receptors but rather by P2Y receptors. The expression of P2Y1, P2Y2, and P2Y4 receptors in porcine cells was further confirmed by western blot (WB) analysis (Figure 2B).

Antagonist experiments were performed to verify that the calcium response was indeed mediated by P2Y receptors and not by other receptor systems that might respond to extracellular nucleotides. Pretreatment with the P2Y1-specific antagonist, MRS2179, reversibly blocked the responses to ATP and ADP but had no effect on the UTP-induced response (Figure 2C,D,E). Application of P2Y receptor agonists after washout of the antagonist resulted in restoration of the calcium response.

### Effects of cellular senescence on P2Y receptors

**Figure 3:**
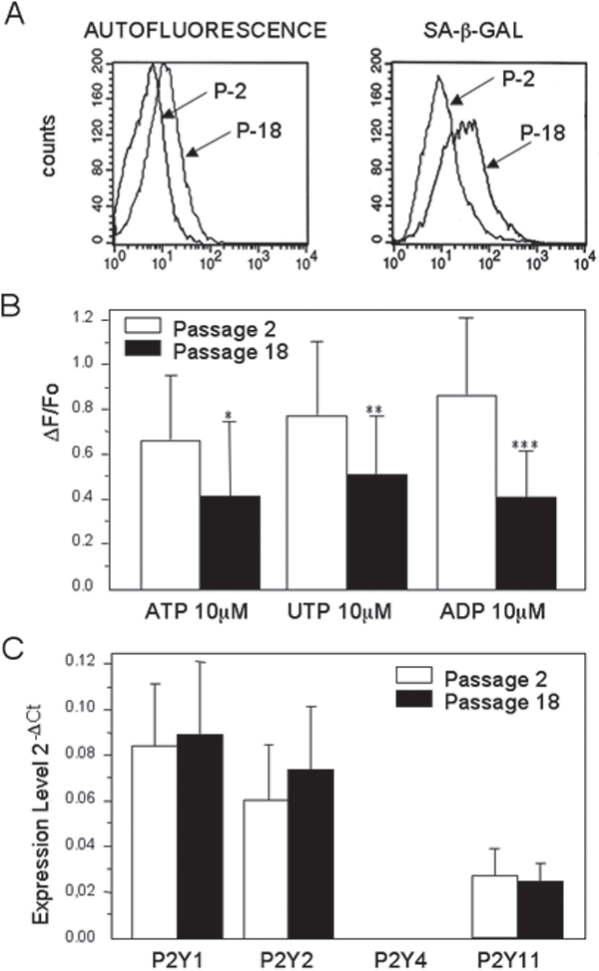
Effect of replicative senescence on P2Y receptor expression and function

TM cells at passage 18 showed a twofold increase in autofluorescence and a threefold increase in sa-β-gal activity (Figure 3A). Together with these features commonly associated with cellular senescence, cells at passage 18 showed a significant decrease in the P2Y receptor-mediated calcium response (ΔF/F_0_) induced by ATP (36%), UTP (34%), and ADP (52%) compared to those at passage 2 (Figure 3B). To determine whether alterations in P2Y receptor responses during cellular senescence could be associated with changes of expression of P2Y receptors and levels of mRNA where analyzed by quantitative RT–PCR. No significant difference in expression between P2Y receptor subtypes were observed between cells at passages 2 and 18. P2Y4 signal was absent from all mRNA samples, but the functionality of the designed primer was verified by detection of signal from genomic DNA (Figure 3C).

The increase of the cellular senescence markers, autofluorescence, and sa-β-gal activity in cells at passage 18 (P-18) compared to cells at passage 2 (P-2) was evaluated by FACS analysis (**A**). The data are representative of three independent experiments. To evaluate whether cellular senescence leads to a decrease in the ability of the TM cells to respond to P2Y agonists, the calcium responses (ΔF/F_0_) to all three agonists were measured in TM cells at passage 18 and compared with cells at passage 2. The decline in ΔF/F_0_ in cells at passage 18 was statistically significant in all cases (The asterisk indicates p=0.03, n=15; the double asterisk means p<0.001, n=30; three asterisks indicate that p=0.001, n=12; **B**). The decrease in ΔF/F_0_ was not associated with any significant decrease in expression of the mRNAs for P2Y1, P2Y2, and P2Y11. The mRNA for P2Y4 receptor subtype was not detected in TM cells. The functionality of our designed primer was verified with signal isolation from a genomic DNA sample. The P2Y11 subtype appeared at a much later PCR cycle, suggesting that it is present in smaller quantities than P2Y1 and P2Y2 receptor subtypes (**C**).

## Discussion

The morphology of the outflow pathway has been demonstrated to change significantly under the influence of variations in IOP [4-9,23]. Increased IOP results in distention and stretching of the outflow pathway (TM) and its individual cells while decreased IOP leads to relaxation of the tissue. Different cell types including those from TM have the ability to sense and respond to mechanical stress by initiating a variety of intracellular signaling mechanisms [24-26]. Therefore, these cellular mechanisms provide a potential means for the TM cells to “sense” changes in IOP and generate homeostatic responses aimed at restoring normal IOP values.

To study the responses of TM cells to mechanical stress, it is important to use experimental systems that model the stress condition of the TM in vivo. So far, most studies of the effects of mechanical stress on TM cells have been performed using static models. These studies have demonstrated cytoskeletal changes, induction of gene expression, and activation of regulatory pathways in response to cell stretching [24,27,28]. However, choroidal expansion and contraction during systole and diastole as well as contraction/relaxation of the ciliary muscle during blinking and eye movement result in repetitive cycling of IOP. These IOP changes of small magnitude likely impose some amount of cyclic mechanical stress on the TM. Therefore, a model of mechanical stress in cell culture based on cyclic stretching might better approximate the true nature of mechanical stress that affects TM cells in vivo. We selected 10% stretching as our initial starting point as this may model the stress levels involved in the outflow pathway [23].

In many physiologic systems, mechanical stress has been shown to induce the release of ATP from intracellular compartments. The released ATP is thought to act on purinergic receptors in an autocrine/paracrine manner on the source tissue to maintain homeostasis. Subepithelial fibroblasts in the gastrointestinal tract act as a mechanosensor by releasing ATP in response to intestinal distension [10]. The released ATP activates P2Y1 receptors, inducing Ca^2+^ wave propagation, which can be transduced from fibroblasts to adjacent neuronal cells and effect peristalsis. In the respiratory system, alveolar type I cells are believed to act as a mechanosensor to release ATP in response to mechanical deformation. Extracellular ATP then acts in a paracrine manner to regulate surfactant secretion from alveolar type II cells [14]. ATP and other purinergic agonists such as UTP modulate the sensitivity of tissues to mechanical stress in several other physiologic systems including airway epithelium, vascular endothelium, mammary alveoli, osteoblasts, and ureter/bladder epithelium [11-13,15].

Our results indicate that mechanical stretch induces ATP release from TM cells. ATP concentrations in the culture medium were significantly higher in mechanically stretched cells compared to unstressed controls. Significant accumulation of ATP in the bulk extracellular compartment was observed only with pharmacological inhibition of endogenous ectonucleotidase activity. ATP released into the extracellular environment may be captured by P2 purinoreceptors at the cell surface of TM cell monolayers or degraded by ecto-ATPases. P2 receptor antagonists such as reactive blue 2 help to inhibit ATP hydrolysis both by occupying ecto-ATPase binding sites and by competing with extracellular ATP for cell surface P2 receptors [29]. We were able to partially inhibit ATP degradation by using a combination of the specific ecto-ATPase inhibitor, ARL-67156, and the P2 receptor antagonist, reactive blue 2. Given the potential for ATP to act in an autocrine/paracrine fashion on functional P2Y receptors on TM cells to decrease outflow resistance, our results are consistent with the hypothesis that ATP may be involved in a homeostatic mechanism that modulates outflow resistance in response to increased IOP.

The accumulation of ATP in the extracellular environment of TM cells reflects the competing rates of ATP release from intracellular sources and ATP hydrolysis by ectonucleotidases. Our experiments demonstrated an additional interesting result: ecto-ATPase activity increases in response to mechanical stress. Ecto-ATPases may be an important mechanism to regulate purinergic signaling in the TM. The rapid degradation of ATP in the absence of ectonucleotidase inhibitors further supports the theory of purinergic signaling as an important IOP regulating pathway. Mechanical stress induces both ATP release and an increase in ecto-ATPase activity, providing a rapid on/off signal for the stimulation of purinergic receptors.

The ATP concentrations that we measured in the bulk extracellular compartment were in the nanomolar range both with and without mechanical stimulation. These concentrations are orders of magnitude lower than the amounts required for threshold activation of P2Y receptors. However, it is unlikely that the ATP concentration in the extracellular media accurately reflect the amounts of ATP that are available in the TM cell surface microenvironment and thus do not reflect the ATP concentration that is available to directly stimulate P2Y receptors on TM cells. First, even with the addition of the specific ecto-ATPase inhibitor, ARL-67156, and the P2 receptor antagonist, reactive blue 2, ATP levels declined after 1 h in mechanically stressed cell cultures. This suggests that the pharmacological inhibition of ATP degradation was incomplete and that measured levels of ATP were lower than actual levels in bathing media. Moreover, released ATP is initially distributed in unstirred layers of bathing media adjacent to the TM cell monolayer surface, precisely where ecto-ATPases are primarily believed to reside. Thus, ATP is degraded rapidly upon release, further supporting the possibility that measured ATP levels were considerably lower than the ATP concentration “sensed” by P2Y receptors. Finally, TM cells in vivo are exposed to a much smaller volume of aqueous humor than the volume of culture medium in experimental conditions. In TM, cells adhere to trabeculae and are exposed to aqueous humor flowing in the small intertrabecular spaces. Moreover, these spaces are believed to be filled with proteoglycans, further reducing the amount of volume for diffusion of released ATP. In contrast, experimental conditions were involved in exposing a monolayer of TM cells to several milliliters of culture medium, thus allowing ATP to be greatly diluted upon diffusion into the bulk extracellular compartment. It has been reported that in astrocyte cell cultures, ATP levels in the extracellular surface microenvironment are considerably higher than the concentrations measured in the bulk culture medium, suggesting that ATP release and hydrolysis occurs in a cell surface microcompartment that is functionally segregated from the bulk extracellular compartment [30]. Although experimental techniques did not allow for the determination of the precise concentrations of ATP near the TM cell surface, it is possible that the colocalization of ATP release sites and ecto-ATPases at the cell surface may act to spatially restrict the actions of extracellular ATP as an autocrine/paracrine signaling molecule in the TM and thus allow ATP to modulate aqueous humor outflow resistance.

P2Y-mediated calcium homeostasis has been linked to mechanisms including cell volume changes and extracellular matrix regulation both of which may affect outflow resistance. P2Y receptors have been implicated in homeostatic TM cell volume regulation, and P2Y agonists have been shown to directly increase outflow facility in organ culture [11,12,15]. Although the precise mechanisms are unknown, P2Y-mediated calcium homeostasis may affect cell volume and extracellular matrix composition and thus modulate aqueous humor outflow resistance in the TM. In response to a hypotonic challenge, which decreases outflow facility presumably by decreasing paracellular fluid flow, P2Y receptors have been reported to mediate the activation of a Ca^2+^-dependent K^+^ channel, leading to a regulatory cell volume decrease [2].

The pharmacology of the P2Y receptor-mediated calcium response in TM cells has not been characterized in porcine TM cells. Previously reported experiments with P2Y receptors in the TM have been performed with bovine tissue; however, the volume and size of the porcine eye is closer to the human eye than any other species. Moreover, the trabecular tissue of the pig eye is more similar to human TM in size, shape, and architecture than other animal models [31]. Thus, we first set out to verify the presence of functional P2Y receptors in this system.

Currently, there is conflicting evidence regarding the specific P2Y receptors involved in the TM cell response to ATP. Crosson et al. reported no P2Y2 receptor expression and no involvement of functional P2Y1 receptors in the ATP-induced intracellular calcium rise in bovine TM cells. The experiments conducted by Soto et al. [2] also in bovine TM cells suggest that ATP may activate the P2Y signaling cascade primarily through P2Y2 receptors with potential contributions from the P2Y1 subtype. The agonist/antagonist response profile in porcine TM cells was consistent with the presence of functional P2Y1 and P2Y2 receptors. Administration of ATP, ADP, and UTP each resulted in a calcium increase in porcine TM cells. UDP, a P2Y6-specific agonist, did not induce a calcium rise, indicating the absence of functional P2Y6 receptors. The P2Y1 receptor responds primarily to adenine nucleotides; inhibition of the ADP-induced response by MRS2179, a specific P2Y1 antagonist, supports the presence of this receptor subtype. The ATP-elicited intracellular calcium rise was blocked by MRS2179, suggesting that ATP also operates primarily through the P2Y1 subtype. ATP is also a known agonist of the P2Y11 receptor subtype for which there is currently no known specific antagonist; thus, we were unable to rule out a contribution from these receptors. The calcium rise induced by UTP is consistent with the presence of functional P2Y2 or P2Y4 receptors. Since specific antagonists of the P2Y2 and P2Y4 receptors have not yet been identified, the contributions of each individual subtype could not be pharmacologically resolved. However, the signal for P2Y4 receptor mRNA was not detected by RT–PCR in porcine TM cells.

The magnitudes of the calcium responses to ATP were not significantly altered after incubation and perfusion in low calcium (EGTA) Ringer's solution. These results suggest that the calcium responsible for the ATP-induced response originates from an intracellular rather than extracellular source. P2X receptors, another class of purinergic receptors, are ATP-gated ion channels that increase cell membrane permeability to extracellular cations including Ca^2+^. However, our data indicate that the calcium response was mediated by P2Y receptors rather than P2X receptors.

Few studies on P2Y receptors in the TM have been reported in the literature. Crosson et al. probed P2Y pharmacology in bovine and human TM cells [32]. Our results demonstrate that porcine TM like bovine and human TM responds to P2Y agonists with an increase in calcium that decreases back to baseline within minutes. The morphology of the calcium response appears to be similar in all three species. However, the specific pharmacology may differ slightly. While Crosson et al. [32] reported that human TM cells have functional P2Y1 and P2Y4 receptors, our results showed an absence of the P2Y4 receptor and the presence of functional P2Y2 receptors in porcine TM.

The observed ability of TM cells to release ATP after mechanical stress and the presence of functional P2Y receptors capable of activating intracellular signaling pathways in response to extracellular ATP and ATP derivatives could potentially contribute to the mechanisms by which TM cells might sense and respond to changes in IOP. The effects of specific P2Y1 agonists and antagonists on the outflow facility of perfused anterior segments from bovine eyes reported by Soto et al. [3] is consistent with the presence of such mechanisms. However, additional experiments in organ culture and in vivo will be necessary to evaluate the relative importance of ATP release and P2Y receptor-mediated responses in the modulation of aqueous humor outflow facility.

One potential factor that could lead over time to the dysregulation of such response mechanism is cellular senescence. We have previously reported a marked increase in features characteristic of cellular senescence in TM cells from old donors as well as an increase of senescent markers in the TM tissue from POAG donors compared to age-matched controls [19]. Based on these observations, we have proposed that similar to other age-related diseases [17,18], accumulation of senescent cells could contribute to the loss of tissue integrity and function in the TM. Senescent cells frequently demonstrate a decreased ability to respond to normal physiologic stimuli including the suppression of calcium-dependent membrane currents, which leads to a generalized inefficiency of calcium-dependent signal transduction pathways [20]. Our results showed that TM senescent cells experienced a statistically significant decrease in the P2Y agonist-induced cytosolic calcium response compared to controls. Since the mRNA levels of P2Y receptor subtypes measured by real-time quantitative RT–PCR did not differ significantly between senescent cells and controls, such decreased response in senescence cells does not appear to result from changes in the expression levels of P2Y receptors. Rather, alterations in the functionality of either P2Y receptor proteins or downstream effectors could contribute to changes in the P2Y-mediated calcium response. Given the implied effects of P2Y receptor activation on outflow physiology, the perturbation of calcium homeostasis in senescent cells could potentially contribute to a decreased capacity to regulate outflow resistance, ultimately resulting in elevated IOP and increasing the risk for glaucoma.

In summary, the ability to release ATP in response to mechanical stress and the presence of functional P2Y receptors in TM cells suggest a novel mechanism by which TM cells could sense and respond to changes in IOP. In addition, the decrease in P2Y receptor-mediated calcium responses observed in senescent TM cells suggests that the dysregulation of calcium homeostasis in senescence may contribute to the alterations of TM in aging and POAG.
